# P-614. Why Aren’t We Treating? Reasons for Antiviral Non-Prescription in Hospitalized Children with Influenza

**DOI:** 10.1093/ofid/ofaf695.827

**Published:** 2026-01-11

**Authors:** Christina B Felsen, Erin Licherdell, Maria Gaitan, Ghinwa Dumyati, Brenda L Tesini

**Affiliations:** University of Rochester School of Medicine and Dentistry, Rochester, NY; University of Rochester Medical Center, Rochester, New York; Rochester Emerging Infections Program, University of Rochester Medical Center, Rochester, New York; New York Emerging Infections Program and University of Rochester Medical Center, Rochester, New York; University of Rochester, Rocheter, New York

## Abstract

**Background:**

Antiviral treatment is recommended for all children hospitalized with influenza to reduce disease severity and complications. However, antiviral use has declined since the COVID-19 pandemic. We aimed to investigate reasons for antiviral non-prescription in children hospitalized with influenza in Western NY.

**Table 1.**
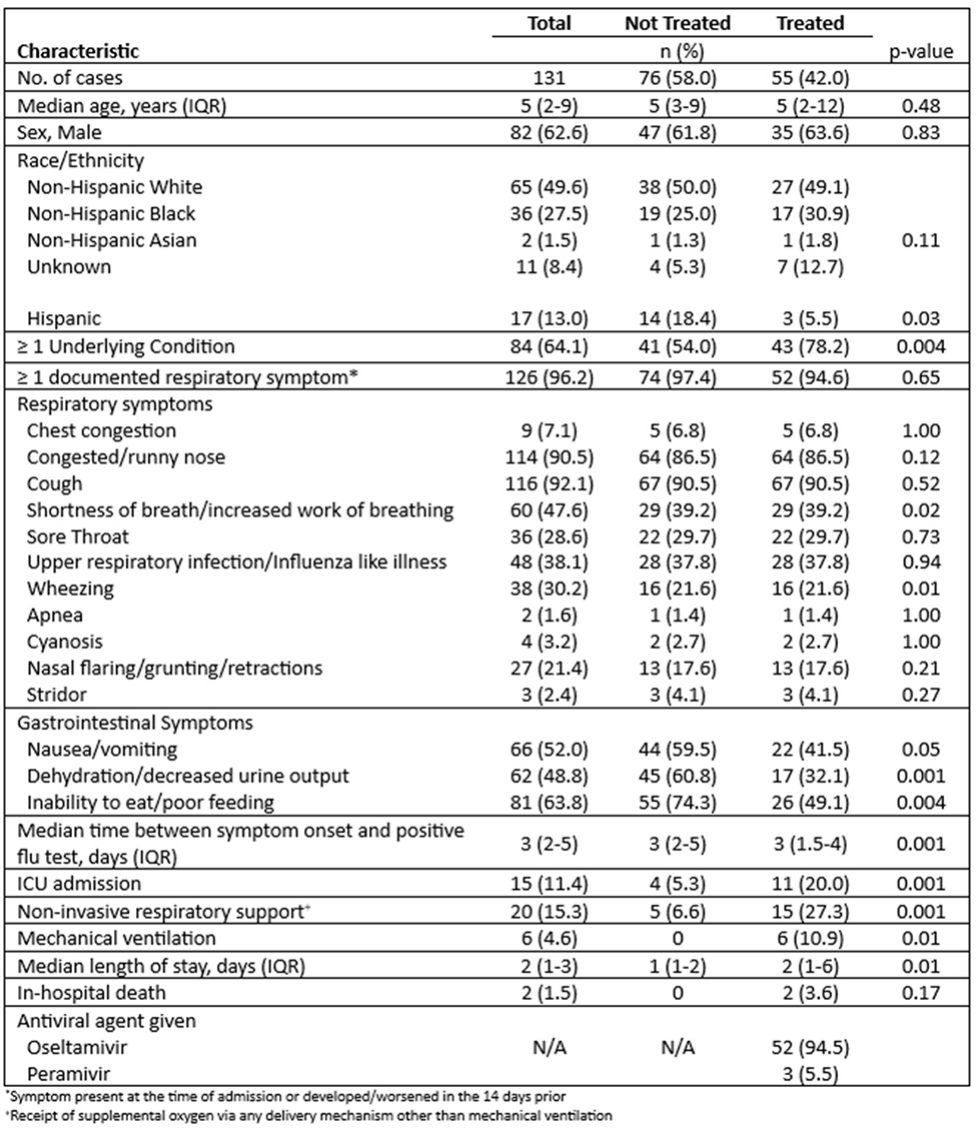
Characteristics of Children Hospitalized with Influenza Stratified by Antiviral Treatment, 2023-2024

**Table 2.**
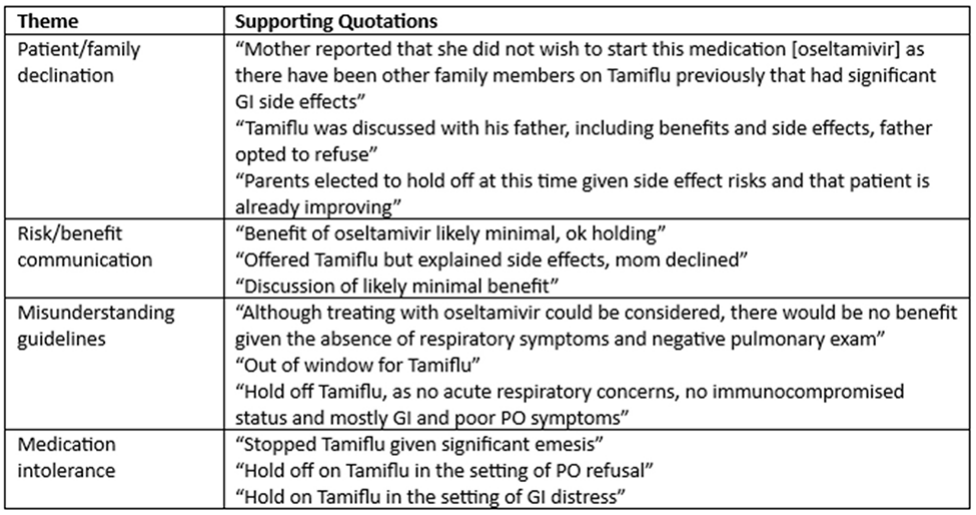
Themes and Associated Quotations: Reasons for Antiviral Non-Prescription

**Methods:**

From 10/2023 to 4/2024, we identified children < 18 years of age hospitalized with a positive, laboratory-confirmed influenza test during or in the 14 days prior to hospitalization in a 7-county catchment area in Western, NY as part of CDC’s FluSurv-NET. Clinical and demographic data were abstracted from medical records and compared across treatment groups. Reasons for antiviral non-prescription were transcribed verbatim and analyzed qualitatively to identify key themes.

**Results:**

131 children were hospitalized with influenza during the surveillance period, of which 76 (58%) did not receive antiviral treatment. Untreated children had a shorter length of stay and were less likely to require ICU admission, non-invasive respiratory support or mechanical ventilation. They were also less likely to have a documented underlying condition. While most (96.2%) children presented with respiratory symptoms, untreated children were more likely to have gastrointestinal symptoms (Table 1). Reasons for antiviral non-prescription were documented for 50% of the untreated children; 4 themes emerged: 1) patient/family declination, 2) risk/benefit communication, 3) misunderstanding guidelines, and 4) medication intolerance. Providers frequently emphasized the risks of antivirals during family discussions and did not adhere to established treatment guidelines, withholding treatment in children with illness duration exceeding 2 days, or lacking high-risk conditions or respiratory symptoms (Table 2).

**Conclusion:**

Over half of the children hospitalized with influenza did not receive antivirals, with treatment more common in children with greater medical complexity. Our findings highlight the need for further education to ensure treatment of all hospitalized children, regardless of duration of illness, comorbidities or symptomology. Additionally, there is a need for guidance on effective communication strategies to emphasize the benefits of antiviral treatment.

**Disclosures:**

Brenda L. Tesini, MD, Merck: Honoraria

